# How Does Adult Temperament Relate to ADHD Symptom Domains? Testing the Dual-Pathway Model

**DOI:** 10.1177/10870547251393062

**Published:** 2025-11-29

**Authors:** Clara Teuchert, Julia Kerner auch Koerner, Monika Daseking, Henning Heinze

**Affiliations:** 1 Helmut-Schmidt-University/University of the Federal Armed Forces Hamburg, Germany; 2 University of Münster, Germany; 3 IDeA - Center for Research on Individual Development and Adaptive Education of Children at Risk, Frankfurt, Germany

**Keywords:** ADHD, temperament, dual-pathway model, effortful control, adult

## Abstract

**Object:**

Temperament provides a valuable framework for understanding ADHD across the lifespan, as extreme temperamental traits are considered etiological risk factors. The dual-pathway model links specific temperamental traits to ADHD symptom domains: elevated reactive traits, surgency and negative affect, to hyperactivity/impulsivity, and a low regulatory trait, effortful control, to inattention.

**Method:**

One hundred fifty-eight adults (79 with clinical diagnoses of ADHD and 79 controls) filled in the Adult Temperament Questionnaire. Consistent with a compensatory extension of the dual-pathway model, it was hypothesized that effortful control would moderate the effects of reactive traits (surgency/negative affect) on hyperactive/impulsive symptoms and influence both ADHD symptom domains. For exploratory purposes, orienting sensitivity, an adult temperament factor related to perceptual sensitivity, was included in the analyses.

**Results:**

Binary logistic regression identified lower effortful control as the strongest predictor of an ADHD diagnosis. Negative affect had a significant but small effect, while surgency and orienting sensitivity were non-significant. Two hierarchical regressions were performed for self-rated symptoms of hyperactivity/impulsivity and inattention. Consistent with a compensatory model, effortful control was significantly related to symptom expression in both ADHD symptom domains. Contrary to expectations, surgency did not explain variance in hyperactivity/impulsivity, and the effect of negative affect was strongly reduced, after effortful control was added to the model. Effortful control did not moderate the effects of surgency and negative affect.

**Conclusion:**

These findings challenge the dual-pathway model and highlight self-regulation deficits over reactive traits in sustaining ADHD in adulthood. They underscore the value of temperament-based approaches for refining diagnosis and developing targeted interventions for adult ADHD.

## Introduction

ADHD is a neurobiological developmental and behavioral disorder with childhood and adolescence onset, which is characterized by three core symptoms: hyperactivity, inattention, and impulsivity (Heine & Exner, 2021). Up to 70% of affected children and adolescents continue to suffer from the associated symptoms into adulthood, resulting in prevalence rates for adult ADHD in Europe of 2% to 5% (de Zwaan et al., 2012; Heine & Exner, 2021). The cognitive-behavioral approach explains the development of ADHD as emerging from negative interactions between neuropsychological predispositions, environmental factors, cognitive and behavioral patterns, as well as adverse learning experiences (Heine & Exner, 2021; Safren et al., 2004). In this context, the psychobiological construct of temperament may help identify intrapersonal risk factors by determining explicit temperamental traits that are associated with an increased likelihood of developing and maintaining ADHD symptoms (Martel et al., 2014).

### Temperament in Adulthood

It is agreed upon that temperament can be seen as a multi-dimensional construct that manifests itself in a person’s reactions and behavior from early childhood (De Pauw & Mervielde, 2010; Kagan & Snidman, 2004; Rothbart & Bates, 2006). As defined by Rothbart and Bates (2006), temperament refers to constitutionally determined individual differences in emotional, motor, and attentional reactivity and self-regulation. These differences become apparent under specific conditions and are assumed to have a genetic and environmental basis (Nigg, 2006). Whereas the dimension of reactivity is further subdivided into the two reactive temperament factors of surgency and negative affect, self-regulation is characterized by the factor of effortful control. Surgency is associated with positive emotionality, increased activity levels, impulsivity, and reward sensitivity, while negative affect is associated with negative emotions such as fear, anger, frustration, and discomfort, as well as sensitivity to punishment (Posner & Rothbart, 2000; Rothbart & Ahadi, 1994). Both reactive factors are measurable from the first year of life and determine intuitive approach and avoidance behavior (Nigg, 2006; Posner & Rothbart, 2000; Rothbart et al., 2000). Effortful control consists of abilities such as attentional focus, error detection, and the use of coping strategies (Posner & Rothbart, 2000). It’s development begins in the third year of life and progresses through pre-school age and adolescence (Rothbart et al., 2006). Effortful control is referred to as a top-down instance of behavioral regulation (Santens et al., 2020), whereas surgency and negative affect are largely automatic, bottom-up processes that are triggered by external or internal stimuli (Posner & Rothbart, 2000). A fourth temperament factor, orienting sensitivity, has been introduced to the adult temperament framework. Orienting sensitivity is related to perceptual sensitivity to low-intensity internal, external, and emotional cues and builds on a network used to focus attention on relevant locations and to register new items (Evans & Rothbart, 2007; Posner & Rothbart, 2000; Wiltink et al., 2006). While these abilities are included in other factors, such as effortful control, in measures for children and adolescents, orienting sensitivity has been described as a separate factor for adults (Ellis, 2002; Posner & Rothbart, 2000). Temperament is thought to emerge through continuous processes of reorganization and adaptation (Kagan & Snidman, 2004), and is shaped by a reciprocal interaction between temperament-related genotypes and environmental influences (Rothbart & Bates, 2006). As a result, the innate response tendencies to positive or negative stimuli observed in early childhood are unlikely to remain unchanged into adulthood. Instead, these early dispositions are gradually transformed through developmental processes such as learning, socialization, and brain maturation (Rothbart & Bates, 2006). Within this developmental trajectory, temperament is considered a foundational substrate for the emergence of personality (Evans & Rothbart, 2007; Ponikiewska et al., 2022). In adulthood, it is conceptualized as a core component of personality, reflecting its neurobiologically grounded affective, activational, and attentional dimensions of reactivity and self-regulation (Evans & Rothbart, 2007; Strelau, 2017). The broader construct of personality builds upon these temperamental foundations and encompasses more complex forms of social adaptation, including internalized values, social norms, cognitive schemas, and habitual behavior patterns (Evans & Rothbart, 2007; Strelau, 2017). Accordingly, temperament and personality represent overlapping yet distinct constructs that should be integrated within a comprehensive biopsychological taxonomy (De Pauw & Mervielde, 2010).

### The Dual-Pathway Model and Compensatory Model of Temperament and ADHD

Traditionally, ADHD research focused on impairments in cold executive functions (Barkley, 1997; Diamond, 2013). Cold executive functions are considered a prerequisite for goal-directed behavior and include inhibitory skills, working memory as well as skills for maintaining attention or cognitive switching. Some of these abilities are also largely associated with effortful control (Jones et al., 2016; Kälin & Roebers, 2021; Tiego et al., 2020; Zelazo, 2020). In addition, the delay aversion hypothesis, which focuses on hot executive functions like motivational control and emotion regulation, has been gaining influence over the last decades (Solanto et al., 2001). It interprets the impulsivity typically seen in ADHD patients as avoidance of reward delay perceived as aversive (Kerner auch Koerner et al., 2018; Solanto et al., 2001). Sensitivity to reward as well as approach/avoidance behavior are considered core features of the reactive factors of temperament (Rothbart & Ahadi, 1994; Rothbart & Bates, 2006).

**Figure 1.**
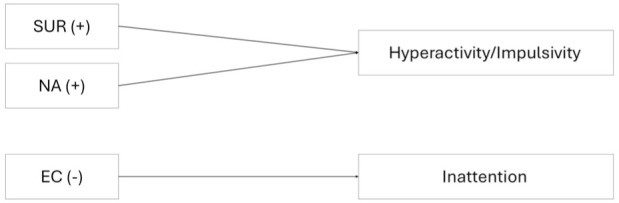
Dual-pathway model of temperament, which links elevations in both reactive traits, negative affect (NA) and surgency (SUR), to an increase in hyperactivity/impulsivity, and deficits in effortful control (EC) to an increase inattention. Thus, a positive association (+) of SUR and NA with hyperactive/impulsive symptoms and a negative association (-) of EC with inattentive symptoms is assumed.

Sonuga-Barke (2002, 2005) integrated both approaches within the dual-pathway model. This model provides an explanatory approach for different symptomatic presentations of ADHD. Since the three core criteria do not necessarily occur together, the DSM-5 distinguishes three predominant presentations of ADHD: the predominantly hyperactive-impulsive or predominantly inattentive presentation, and the mixed presentation (American Psychiatric Association, 2013). The dual-pathway model identifies impaired cold executive functions as the main cause of inattentive symptoms (Martel et al., 2009; Sonuga-Barke, 2005), whereas motivational deficits are associated with hyperactive and impulsive behavior (Martel et al., 2009; Rubia, 2018; Sonuga-Barke, 2005). The model was supported by findings showing that both traits contributed independently to ADHD symptoms (Nigg et al., 2005; Thorell, 2007). Nigg et al. (2004) applied the dual-pathway model to temperament research (Martel et al., 2009; Martel & Nigg, 2006; Nigg, 2006). They mapped both superordinate dimensions of self-regulation and reactivity to the pathways proposed by Sonuga-Barke (2005), linking the primarily inattentive subtype to deficits in effortful control and the primarily hyperactive-impulsive subtype to increments in surgency and negative affect (see Figure 1; Martel et al., 2009; Martel & Nigg, 2006; Nigg, 2006).

**Figure 2.**
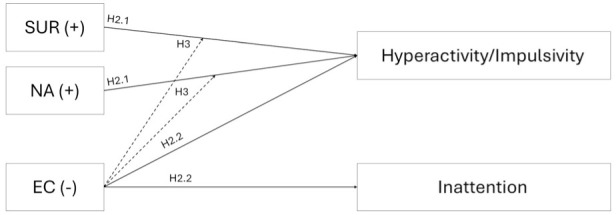
Compensatory model of temperament and ADHD, which links elevations in both reactive traits, negative affect (NA) and surgency (SUR), to an increase in hyperactivity/impulsivity (H2.1, solid arrows), and deficits in effortful control (EC) to an increase in both ADHD symptom domains (H2.2, solid arrows). Additionally, the model proposes a moderating (protective) effect of EC (H3, dotted arrows) on SUR and NA.

However, an additional direct link between effortful control and hyperactive/impulsive symptoms can be hypothesized. Effortful control encompasses an aspect referred to as inhibitory control, which includes the ability to suppress or regulate emotionally charged impulses (De Pauw & Mervielde, 2011; Gomez et al., 2014; Martel & Nigg, 2006; Wiltink et al., 2006). Jones et al. (2016) also highlight that effortful control incorporates more complex skills such as delay of gratification, motivation and willpower, and is therefore likely to be involved in motivational response systems. Furthermore, Martel et al. (2009) suggest that ADHD symptoms may result not only from direct pathways but also from their interaction, with effortful control acting as a potential protective factor. High effortful control may reduce, and low effortful control may amplify, the impact of reactive temperament. Thus, the extended compensatory dual-pathway model incorporates a direct impact of effortful control on both ADHD symptom domains, as well as a moderating influence on reactive traits (see Figure 2).

Although the theoretical framework of the dual-pathway model does not include orienting sensitivity, the factor may play a role in attentional processes as it captures differences in detecting subtle or peripheral stimuli (Evans & Rothbart, 2007). Consistent with this idea, Gomez et al. (2014) demonstrated significant associations between adult ADHD and a specific subscale of orienting sensitivity, suggesting that the factor could contribute to core ADHD symptoms.

There is empirical evidence for both the dual-pathway model (Kerner auch Koerner et al., 2018; Martel et al., 2009) and compensatory extensions of the model (Martel & Nigg, 2006; Rabinovitz et al., 2016) in childhood and adolescence. Furthermore, research indicates that children and adolescents with ADHD typically exhibit lower effortful control and higher surgency and/or negative affect compared to healthy controls (Kerner auch Koerner et al., 2018; Krieger et al., 2019; Martel et al., 2009, 2014; Martel & Nigg, 2006). Although significant associations between temperament traits and ADHD symptom domains have been demonstrated in the childhood literature, only one adult study has examined the relationship between Rothbart’s temperament factors and ADHD symptoms in an adult, non-clinical sample (Gomez et al., 2014). The main findings showed an association between a specific inattention factor with low effortful control, while a specific hyperactivity/impulsivity factor was associated with reduced inhibitory control (a subconstruct of effortful control) and a tendency toward high sociability (a subconstruct of surgency). A general ADHD factor was associated with low effortful control, high negative affect and high associative sensitivity (a subscale of orienting sensitivity; Gomez et al., 2014). To our knowledge, the relationship between Rothbart’s temperament factors and adult ADHD has not been examined in a clinical sample or contrasted with a control group. The present study aims to fill this gap and to identify intrapersonal temperament factors associated with the persistence of an ADHD diagnosis into adulthood.

Our primary question was whether temperamental traits influence the likelihood of being diagnosed with ADHD in adulthood. It was expected that high levels of surgency and negative affect would increase the odds of being classified in the ADHD group (**H1.1**), whereas effortful control would decrease this probability (**H1.2**). To further elucidate the link between orienting sensitivity and adult ADHD, the factor was included in our analyses in an exploratory manner. Furthermore, it was examined whether surgency and negative affect exclusively explain variance in hyperactive/impulsive symptoms and whether effortful control exclusively explains variance in inattentive symptoms, in line with the dual-pathway model. In accordance with the compensatory model of ADHD, it was hypothesized that surgency and negative affect would only be associated with hyperactive/impulsive symptoms (**H2.1**), whereas effortful control would predict both symptom domains (**H2.2**; see Figure 2). It was also hypothesized that the effect of surgency and negative affect on hyperactive/impulsive symptoms would be moderated by effortful control (**H3**, see Figure 2, dotted arrow).

## Method

### Sample and Procedure

The study was approved by an internal data protection and ethics committee and was carried out in cooperation with several outpatient ADHD clinics in Hamburg, Germany. A clinical sample of ADHD patients was recruited, while the comparison sample was selected to exclude undiagnosed ADHD cases. The diagnostic assessment included multiple self-report questionnaires, a standardized diagnostic interview, cognitive performance tests, external assessment through school reports and family members, and behavioral observations during all testing. The diagnostic decision was based on the DSM-5 criteria and made in accordance with clinical guidelines, with primary emphasis on the structured clinical interview and integration of all additional information sources. Interviews were headed by a trained psychologist with clinical experience in adult ADHD. In the case of a clear diagnosis of ADHD, patients were enrolled in the ADHD study group and informed about further medical and therapeutic treatment options by the responsible treatment providers. If the clinical impression was ambiguous, additional differential diagnosis was recommended. In the case of a clear negative result, the persons data were added to the potential pool for a control group. There were no cases of psychostimulant prescription in the ADHD group, as subjects did not have an established diagnosis of ADHD at the time of data collection. The control group sample was additionally recruited through an internal call via a university’s student network. From a pool of 102 individuals, those who best matched the clinical sample in terms of both age and gender were selected for the present study to minimize the influence of these potential confounding factors. To ensure the absence of undetected ADHD cases in the control group, a cut-off criterion was applied: participants with *T*-scores ≥65 on either the DSM-ADHD scale or the ADHD Index scale of the ADHD assessment tool were excluded. Sociodemographic data for the two samples are presented in Table 1. The sample consisted of a total of 158 participants. The mean age of the ADHD patients was 31.33 years (*SD* = 9.50), compared to 27.58 years (*SD* = 10.29) in the control group. Most participants in both groups were male (63.3%; *n* = 50). A university entrance degree was held by 54 ADHD patients (72.0%) and 70 controls (89.7%).

**Table 1.** Demographic Description of the Sample by Group.

Variable	ADHD group (*n* = 79)	Control group (*n* = 79)	Group difference
Gender (*n* and % of women)	29 (36.7)	29 (36.7)	
*M*_age_ (*SD*)	31.33 (9.50)	27.58 (10.29)	.019*^a^
Country of birth Germany	73 (92.4%)	78 (98.7%)	.093^b^
Highest school certificate	(*n* = 75)	(*n* = 78)	<.005*^b^
Intermediate secondary school certificate or lower	21 (28.0%)	8 (10.3%)	
University entrance degree	54 (72.0%)	70 (89.7%)	

*Note.* In Germany, students are placed into different secondary school tracks based on academic performance. “Intermediate secondary school certificate or lower” refers to qualifications obtained from Hauptschule (lower secondary school) or Realschule (intermediate secondary school); “University entrance degree” refers to the Abitur (general higher education entrance qualification). *M* = mean; *SD* = standard deviation; *p < .05.

a *p*-value of *t*-test.

b *p*-value of chi-square test.

### Measurements

#### Adult Temperament

The German version of the short form of the Adult Temperament Questionnaire by Wiltink et al. (2006) was used to assess adult temperament dimensions. The short form of the German Adult Temperament Questionnaire is a 77-item self-report questionnaire, that collects temperament-related preferences and behaviors (Evans & Rothbart, 2007; Rothbart et al., 2000). It measures the four temperament factors of surgency, negative affect, effortful control and orienting sensitivity which are each composed of three to four subscales. Surgency consists of the subscales sociability, high-intensity pleasure, and positive affect. Negative affect comprises the four subscales fear, sadness, discomfort, and frustration. The factor effortful control includes the three subscales inhibitory control, activation control, and attentional control, whereas the factor orienting sensitivity comprises the subscales neutral perceptual sensitivity, affective perceptual sensitivity, and associative sensitivity (Wiltink et al., 2006). The items of the Adult Temperament Questionnaire are answered on a seven-point Likert scale ranging from 1 (strongly disagree) to 7 (strongly agree). Means are calculated for each subscale and the superordinate temperament factors, with higher scores indicating a higher trait expression. The questionnaire was specifically developed to assess enduring temperamental tendencies in adults and has demonstrated convergent validity with broader personality constructs (Evans & Rothbart, 2007; Ponikiewska et al., 2022). The German Adult Temperament Questionnaire was validated by Wiltink et al. (2006) based on a total sample of 329 subjects, consisting of a clinical and a student sub-sample. Construct validity was demonstrated by significant positive correlations with convergent constructs of the NEO Five-Factor Inventory (Borkenau & Ostendorf, 2008). All Cronbach’s alpha coefficients were calculated using data from the current study. For the four main scales, internal consistency ranged from α = .76 to .87, indicating acceptable to good reliability.

#### ADHD Symptoms

The German adaptation of the Conners’ Adult ADHD Rating Scales (CAARS) was used to assess the severity of ADHD symptoms (Christiansen et al., 2014; Conners et al., 2002). The long version of the self-report questionnaire contains 66 items and a total of eight scales. Three scales cover the DSM-4 symptomology and are labeled inattention (DSM-IA), hyperactivity/impulsivity symptoms (DSM-HY/I), and total symptoms of ADHD. In addition, there are four factor-analytically determined scales that assess central ADHD symptoms and behaviors: inattention/memory problems, hyperactivity/motor restlessness, impulsivity/emotional lability and self-concept problems. The scale ADHD Index consists of those items that have the highest discriminatory power between ADHD patients and controls. Items are answered on a 4-point Likert scale ranging from 0 (not at all/never) to 3 (very strong/very often). Age- and gender-specific norms are available (Christiansen et al., 2014). *T*-scores between 60 and 64 are considered marginal, *T*-scores between 65 and 69 are considered severe, and *T*-scores ≥70 are considered very severe. To avoid redundancy in results and multiple testing, the current study focused on the DSM symptom scales to assess associations between temperament factors and ADHD symptoms. Cronbach’s alpha for these scales varied between α = .83 and .92, which can be classified as good to very good.

#### Methodological Approach to Treatment of Item Overlap

The problem of item overlap between temperament and ADHD instruments has been described in previous studies (Nigg, 2006; Wichstrøm et al., 2018). It is evident when comparing the item wordings of the Conners’ Adult ADHD Rating Scales and the Adult Temperament Questionnaire, although the measures used in this study come from different areas of psychological research. This may result in item contamination and an overestimation of effect sizes (Nigg, 2006). Therefore, it was decided that items of the Adult Temperament Questionnaire with literal overlap with the DSM scales of the Conners’ Adult ADHD Rating Scales and DSM-5 diagnostic criteria would be excluded from regression analyses to avoid inflated effect sizes. The decision-making process involved a thorough comparison of the relevant items of the Adult Temperament Questionnaire with the DSM scales and DSM-5 criteria for ADHD. The aim was to strike a balance between the risk of inflating effect sizes and compromising construct validity (Sanson et al., 1990). The latter was ensured by retaining items of the Adult Temperament Questionnaire that demonstrated content overlap only with one of the four factor-analytically determined scales of the Conners’ Adult ADHD Rating Scales. After careful consideration, items from the Adult Temperament Questionnaire describing attentional deficits under emotional activation (e.g., “It is very hard for me to focus my attention when I am distressed”) were retained despite some degree of literal overlap. These items were not considered redundant because they reflect the ability to maintain attentional focus in emotionally salient situations, which is a core component of effortful control (Jones et al., 2016). In this context, effortful control is understood as the capacity to deliberately regulate attention despite emotional distraction, distinguishing it from more general attentional deficits (American Psychiatric Association, 2013; Jones et al., 2016). In contrast, the DSM-5 criteria for inattention describe persistent difficulties in sustaining attention, organizing tasks, and completing activities across a range of contexts, regardless of emotional state. They reflect a broader, context-general attentional impairment rather than the targeted regulatory capacity captured by effortful control (American Psychiatric Association, 2013). In total, 5 of 19 items from the effortful control scale, 2 of 25 items from the negative affect scale, and 1 of 17 items from the surgency scale were excluded. Table A1 in the Appendix provides a detailed overview of all excluded items, and Table A2 lists all items with potential content overlap that were not excluded from the calculations and the respective reasons for non-exclusion. After removing overlapping items from the Adult Temperament Questionnaire, Cronbach’s alpha remained within the range of α = .76 to .87 for the main scales.

### Data Analysis

Data analysis was performed using IBM SPSS Statistics (28.0.1.1). The analysis showed that a total of 0.37% of the questionnaire data were missing. Fifty-nine out of 143 items (41.26%) were affected by missing values and 39 (25.49%) out of 158 cases had at least one missing variable. Littles MCAR test and visual analysis indicated that all data were missing completely at random (χ^2^(4,928) = 4,908.64, *p* = .575). Five-iteration multiple imputation was performed to handle the missing values. All subsequent analyses were based on the combined imputed data. The descriptive analyses involved a demographic comparison (Table 1) as well as descriptive statistics and pairwise correlations of temperament measures (Table 2). To control for alpha error accumulation in the correlational analyses, the significance level was adjusted to *p* < .008 using a Bonferroni correction for six tests; Table 2). Additionally, independent-samples *t*-tests were conducted to compare temperament factors between the ADHD and control groups (see Table 3), enabling a direct comparison with prior findings and effect sizes from an adolescent sample (Krieger et al., 2019). To rule out potential confounding effects of age and education, a subsequent analysis of covariance (ANCOVA) was conducted. The adjusted means and effect sizes are displayed in the appendix (Table A3). A binary logistic regression was performed to assess the possible influence of surgency, negative affect, effortful control and orienting sensitivity on the likelihood of being diagnosed with ADHD (**H1**). All predictors were mean centered to ensure content-based analysis of effects (Dalal & Zickar, 2012). Age and highest school certificate were included as possible confounding factors. To test our **H2.1** and **H2.2**, two hierarchical multiple regressions were conducted with self-rated hyperactive/impulsive symptoms (CAARS DSM-HY/I *T*-scores, Table 5, Steps 1–4) and inattentive symptoms (CAARS DSM-IA *T*-scores, Table 5, Steps 1–4) as the outcome variables. In the first step, the variables age, gender, and highest school certificate were included in both models, to control for these potentially confounding factors. To predict hyperactive/impulsive symptoms, surgency was added to the model in the second step, followed by negative affect and effortful control in the third and fourth steps, respectively, to determine a significant increase in explained variance. To predict inattentive symptoms, effortful control was added to the model in the second step, followed by surgency and negative affect in the third and fourth steps. The moderation hypothesis (**H3**) was examined by adding interaction terms to the hierarchical regression model for predicting hyperactive/impulsive ADHD symptoms (*T*-scores of CAARS scale DSM-HY/I) to simultaneously test both potential moderating effects of effortful control on negative affect and on surgency (Table 5, Step 5). To assess the robustness of the findings, an additional post hoc moderation analysis was performed using the PROCESS macro (version 4.0; Hayes, 2022) for SPSS. This procedure was done only for hyperactive/impulsive symptoms, as neither the compensatory nor the original dual-pathway model predict an association between reactive temperament factors (surgency and negative affect) and inattentive ADHD symptoms (see Figures 1 and 2).

**Table 2.** Descriptives and Pairwise Correlations of Temperament Measures.

Variable	*M (SD)*	Range	SUR	NA	EC
SUR	4.52 (0.84)	1.71–6.59			
NA	3.90 (0.90)	1.54–6.15	−.29**		
EC	3.84 (1.15)	1.68–7.00	.12	−.67**	
OS	4.60 (0.90)	2.60–6.80	.17	.28**	−.12

*Note.* The alpha level was adjusted for multiple comparisons using a Bonferroni correction based on six tests. SUR = surgency; NA = negative affect; EC = effortful control; OS = orienting sensitivity; *M* = mean; *SD* = standard deviation; **p < .001.

**Table 3.** Means, Standard Deviations, and Effect Sizes for Group Differences in Temperament Between ADHD and Control Groups.

	ADHD group (*n* = 79)	Control group (*n* = 79)	*T*	*df*	*p*	*d*
Variable	*M (SD)*	*M* (*SD*)				
Surgency	4.48 (0.88)	4.56 (0.81)	0.554	156	.580	0.088
Negative affect	4.39 (0.69)	3.40 (0.81)	8.240	156	<.001	1.311
Effortful control	2.99 (0.69)	4.68 (0.85)	−13.684	156	<.001	−2.177
Orienting sensitivity	4.76 (0.97)	4.44 (0.79)	2.322	156	.022	0.369

*Note*. ATQ = Adult Temperament Questionnaire; *M* = mean; *SD* = standard deviation; *t* = *t*-value; *df* = degrees of freedom; *p* = *p*-value; *d* = Cohen’s *d*.

## Results

Descriptive statistics and intercorrelations of the temperament variables are provided in Table 2. Group differences in temperament and the corresponding effect sizes are summarized in Table 3. Groups did not differ in self-reported surgency. However, compared to the control group, the ADHD group rated themselves as having significantly higher negative affect and orienting sensitivity and significantly lower effortful control. Cohen’s *d* was 1.31 for negative affect and −2.18 for effortful control, both indicating large effects, whereas the effect for orienting sensitivity was small (*d* = 0.37). The adjusted means and effect sizes from the ANCOVA were comparable to those from the independent-samples *t*-tests, indicating that controlling for age and education did not alter the pattern of self-reported group differences. All estimated coefficients and corresponding odds ratios from the binary logistic regression analysis used to predict an ADHD diagnosis (**H1.1/H1.2**) are reported in Table 4. The omnibus test indicated that the model was statistically significant (*χ^2^*(6) = 119.157, *p* < .001). The Hosmer-Lemeshow test showed a good model fit, and the explained variance was high with Nagelkerke’s *R*^2^ = .71 (Backhaus et al., 2006). Negative affect showed a small to moderate positive effect that reached statistical significance (OR = 2.29, 95% CI [1.05, 4.99], *p* = .038). However, the lower bound and width of the confidence interval warrant cautious interpretation. The effect of surgency was not significant (*p* = .06). Thus, **H1.1** received only partial support. Effortful control showed a strong negative effect (OR = 0.07, 95% CI [0.03, 0.19], *p* < .001), fully supporting **H1.2**.

**Table 4:** Binary Logistic Regression Predicting ADHD Diagnosis.

Variable	*β*	*SE β*	*Wald’s χ^2^*	*df*	*p*	*eβ*	95% confidence interval for *eβ* [lower value, upper value]
SUR	0.649	0.345	3.539	1	.060	1.913	[0.973, 3.760]
NA	0.827	0.398	4.315	1	.038	2.287	[1.048, 4.991]
EC	−2.600	0.477	29.664	1	<.001	0.074	[0.029, 0.189]
OS	0.115	0.315	0.133	1	.715	1.122	[0.605, 2.080]
Age	0.044	0.026	2.891	1	.089	1.045	[0.993, 1.099]
HSC	−0.777	0.606	1.645	1	.200	0.460	[0.140, 1.507]

*Note.* SUR = surgency; NA = negative affect; EC = effortful control; OS = orienting sensitivity; HSC = highest school certificate; *β* = unstandardized regression coefficient; *SE β* = standard error of beta; *eβ* = odds ratio; *Wald’s χ^2^* = Wald chi-square statistic; *df* = degrees of freedom; *p* = *p*-value.

The results of the two hierarchical regressions testing our **H2.1** and **H2.2** are presented in Tables 5 and 6 (Steps 1–4). In the model predicting self-reported hyperactive/impulsive symptoms (CAARS DSM-HY/I *T*-scores), age was a significant confounder, with 6.2% of explained variance in the first step, whereas surgency did not contribute to the explanation of variance when entered in the second step. When negative affect was added in the third step, it emerged as a positive predictor, accounting for an additional 27.5% of variance explained. However, after effortful control was added in the fourth step, the total explained variance increased to 51.2%, and the effect of negative affect dropped substantially from β = .615 (*p* < .001) to β = .188 (*p* = .032), indicating that effortful control may account for a substantial portion of the association between negative affect and self-reported hyperactive/impulsive symptoms. In the model predicting self-reported inattentive symptoms (CAARS DSM-IA *T*-scores), age was again a significant confounding factor, with 4.6% of variance explained in the first step. As hypothesized, effortful control was a significant negative predictor in the second step, with the model accounting for 64.4% of the variance. Neither surgency nor negative affect explained additional variance when added in the third and fourth steps. Thus, effortful control remained the sole significant predictor in the final model, accounting for 65.4% of the variance. As the effects of the reactive factors (surgency and negative affect) on both ADHD symptom domains were either non-significant or markedly reduced by effortful control, **H2.1** was not supported. In contrast, effortful control significantly predicted both self-reported hyperactive/impulsive and inattentive symptoms, offering strong support for **H2.2**.

**Table 5.** Hierarchical Regression with Hyperactive/Impulsive ADHD (CAARS DSM HY/I) Symptoms as Dependent Variables and Temperament (ATQ) as Predictor.

Variable	*B*	*SE (B)*	*β*	*T*	*p*	*R^2^*	*ΔR^2^*
Step 1						.062	—
Age	0.354	0.125	.224	2.840	.005		
HSC	−3.960	2.441	−.132	−1.622	.107		
Gender	2.095	2.541	.066	.824	.411		
Step 2						.059	.003
Age	0.370	0.127	.234	2.919	.004		
HSC	−4.156	2.460	−.138	−1.690	.093		
Gender	2.241	2.553	.070	0.878	.381		
SUR	1.061	1.464	.058	0.725	.470		
Step 3						.337	.275**
Age	0.362	0.106	.229	3,403	<.001		
HSC	−4.349	2.065	−.145	−2,106	.037		
Gender	−5.235	2.335	−.165	−2,242	.026		
SUR	4.813	1.314	.262	3,662	<.001		
NA	10.743	1.332	.615	8,065	<.001		
Step 4						.512	.173**
Age	0.291	0.092	.184	3.169	.002		
HSC	−2.071	1.797	−.069	−1.152	.251		
Gender	−2.627	2.033	−.083	−1.292	.198		
SUR	3.539	1.140	.193	3.104	.002		
NA	3.291	1.518	.188	2.169	.032		
EC	−8.112	1.087	−.572	−7.459	<.001		
Step 5						.506	.001
Age	0.291	0.092	.185	3.157	.002		
HSC	−2.082	1.817	−.069	−1.146	.254		
Gender	−2.552	2.057	−.080	−1.241	.217		
SUR	3.510	1.149	.191	3.054	.003		
NA	3.309	1.528	.189	2.166	.032		
EC	−8.076	1.106	−.569	−7.303	<.001		
ECxSUR	−0.480	1.118	−.025	−0.430	.668		
ECxNA	−0.120	0.810	−.009	−0.148	.883		

*Note.* ATQ = Adult Temperament Questionnaire; CAARS = Conners’ Adult ADHD Rating Scales; DSM HY/I = CAARS DSM scale for hyperactive/impulsive symptoms; SUR = surgency; NA = negative affect; EC = effortful control; HSC = highest school certificate. ECxSUR = interaction/moderating effect of effortful control on surgency; ECxNA = interaction/moderating effect of effortful control on negative affect; *B* = unstandardized regression coefficient; *SE(B)* = standard error of *B; β* = standardized regression coefficient; *t* = *t*-value; *p* = *p*-value; *R*^2^ = coefficient of determination; Δ*R*^2^ = change in *R*^2^; **p < .001.

**Table 6.** Hierarchical Regression with Inattentive ADHD (CAARS DSM IA) Symptoms as Dependent Variables and Temperament (ATQ) as Predictor.

Variable	*B*	*SE (B)*	*β*	*T*	*p*	*R^2^*	*ΔR^2^*
Step 1						.046	—
Age	0.324	0.138	.186	2.342	.020		
HSC	−4.231	2.711	−.128	−1.561	.121		
Gender	3.281	2.823	.094	1.162	.247		
Step 2						.644	.589**
Age	0.177	0.085	.102	2.079	.039		
HSC	−0.604	1.671	−.018	−0.361	.718		
Gender	−0.910	1.744	−.026	−0.522	.603		
EC	−12.313	0.764	−.788	−16.116	<.001		
Step 3						.651	.009*
Age	0.149	0.085	.086	1.742	.084		
HSC	−0.293	1.662	−.009	−0.176	.861		
Gender	−1.118	1.730	−.032	−0.646	.519		
EC	−12.119	0.763	−.775	−15.892	<.001		
SUR	−1.992	0.990	−.098	−2.011	.046		
Step 4						.654	.006
Age	0.157	1,085	.090	1.845	.067		
HSC	−0.611	1,666	−.018	−0.367	.714		
Gender	−2.336	1,885	−.067	−1.239	.217		
EC	−11.067	1,008	−.708	−10.978	<.001		
SUR	−1.385	1,057	−.068	−1.310	.192		
NA	2.231	1,407	.116	1.586	.115		

*Note.* ATQ = Adult Temperament Questionnaire; CAARS = Conners’ Adult ADHD Rating Scales; DSM IA = CAARS DSM scale for inattentive symptoms; SUR = surgency; NA = negative affect; EC = effortful control; HSC = highest school certificate. ECxSUR = interaction/moderating effect of effortful control on surgency; ECxNA = interaction/moderating effect of effortful control on negative affect; *B* = unstandardized regression coefficient; *SE(B)* = standard error of *B; β* = standardized regression coefficient; *t* = *t*-value; *p* = *p*-value; *R*^2^ = coefficient of determination; Δ*R*^2^ = change in *R*^2^; *p < .05; **p < .001.

**Figure 3.**
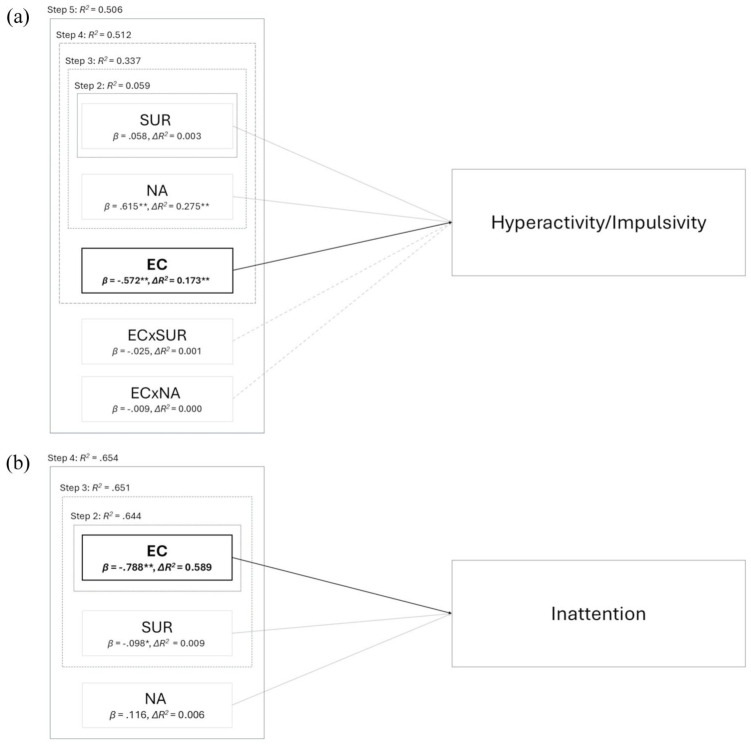
(a) Model for hyperactive/impulsive symptoms derived from hierarchical regression: Step 1 controlled for the potential confounding variables age, gender and highest school certificate and explained 6.2% of variance in hyperactive/impulsive symptoms, while only age was a significant positive predictor (*β* = .224, *p* = .005). To maintain readability, control variables were excluded from the figure. The effect of surgency (SUR) was not significant (Step 2). The effect of negative affect (NA; Step 3) dropped substantially to *β* = .188 (*p* = .032) after effortful control (EC) was added to the model (Step 4). EC significantly contributed to the explanation of variance in hyperactive/impulsive symptoms but there was no moderating effect of EC on SUR (ECxSUR) and NA (ECxNA; Step 5). (b) Model for inattentive symptoms derived from hierarchical regression: Step 1 controlled for the potential confounding variables age, gender and highest school certificate and explained 4.6% of variance in inattentive symptoms, while only age was a significant positive predictor (*β* = .186, *p* = .020). Effortful control (EC) significantly contributed to the explanation of variance in inattentive symptoms (Step 2). Neither SUR nor NA explained additional variance in inattentive symptoms when added in the third and fourth steps. *β* = standardized regression coefficient; *ΔR^2^* = change in explained variance by the specific temperament factor; *R^2^* = amount of variance explained by the model in each step; *p < .05; **p < .001.

None of the interaction terms between effortful control and surgency or negative affect were significant in predicting self-reported hyperactive/impulsive symptoms (CAARS DSM-HY/I scale), leading to the rejection of our **H3** (see Table 5, Step 5). To test the robustness of these findings, a separate examination of the moderation effects was conducted using PROCESS (Model 1; 5,000 bootstrap samples), which also did not yield significant results. Figure 3a and b illustrate the final model of the relationship between temperament factors and ADHD symptom domains derived from the data.

## Discussion

This study investigated the role of temperament in adult ADHD and examined its contribution to diagnostic status and to self-reported symptom expression by testing the dual-pathway model and a compensatory model. While our findings partially align with existing studies, they also underscore notable differences in temperament-ADHD associations in adulthood compared to those reported in childhood and adolescence literature.

### Group Differences in Temperament

The results of the analysis of group differences indicated that, compared to their peers, the ADHD group perceived themselves as having significantly higher negative affect and orienting sensitivity, and significantly lower effortful control. No significant group differences emerged in self-reported surgency. These findings are comparable to those of studies in childhood and adolescence that have used related instruments from the Rothbart temperament framework, such as the Children’s Behavior Questionnaire (Putnam & Rothbart, 2006) and the Early Adolescent Temperament Questionnaire (Ellis & Rothbart, 2001). The absence of significant group differences in self-reported surgency contradicts previous research in younger populations, where elevated informant-reported surgency is considered a key risk factor for developing ADHD and a distinguishing characteristic between groups (Kerner auch Koerner et al., 2018; Krieger et al., 2019; Martel et al., 2014). However, our findings align with prior studies reporting no differences in self-reported extraversion between adults with and without an ADHD diagnosis (Nigg et al., 2002). Extraversion, a personality trait reflecting sociability, outgoingness, optimism, and sensation seeking, is strongly associated with the adult surgency factor (Evans & Rothbart, 2007). While in childhood and adolescence surgency is linked to high-intensity pleasure, elevated activity levels, impulsivity, and approach-related aspects of shyness (Ellis & Rothbart, 2001; Putnam & Rothbart, 2006), its manifestation in adulthood indicates a developmental shift. In adults, surgency continues to capture high-intensity pleasure, but places less emphasis on components such as activity level and impulsivity, instead reflecting aspects such as sociability and positive emotionality (Evans & Rothbart, 2007; Wiltink et al., 2006). These observations suggest that ADHD-related group differences in childhood surgency may be primarily driven by components such as high motor activity and impulsivity. This assumption is further supported by findings from a meta-analysis by Gomez and Corr (2010), which showed no or minimal group differences in positive emotionality between children with ADHD and community samples. The moderate negative correlation between self-reported surgency and negative affect in our sample (see Table 2) further suggests that adults who experience negative affective states more frequently may tend to evaluate themselves as less sociable or less prone to positive affect. This could contribute to a lowered self-perception of surgency in populations with adult ADHD.

The comparison of effect sizes for self-reported negative affect (*d* = 1.31) and effortful control (*d* = −2.18) in our adult sample with those for parent-reported temperament in Krieger et al.’s (2019) adolescent sample (*d* = 0.81 for negative affect; *d* = −2.23 for effortful control) suggests a developmental pattern: Individuals with ADHD may experience an increase in negative affect over time, while self-regulatory deficits seem to represent an enduring core feature of persistent ADHD. While it is conceivable that the emotional consequences of impaired effortful control may become more pronounced with advancing age, another possible explanation for the increasing divergence in self-perceived negative affect could lie in the cumulative exposure to adverse life events and greater difficulties during life transitions that individuals with ADHD often face (LaCount et al., 2019). Concurrently, individuals without ADHD may develop and refine more effective coping strategies for managing negative affective states during the transition from adolescence to adulthood, thereby further amplifying group differences in self-reported emotional functioning (Retz et al., 2012; Seymour et al., 2019). This interpretation is supported by the strong negative correlation between negative affect and effortful control observed in our data (see Table 2). Self-perceived deficits in attentional, inhibitory, and activational control may intensify the experience of negative affective states such as frustration or fear, which in turn may prevent the effective use of effortful control strategies (Gomez & Corr, 2010; Rabinovitz et al., 2016; Scime & Norvilitis, 2006).

The ADHD group’s higher mean score on the orienting sensitivity scale suggests that individuals with ADHD perceive themselves as more sensitive to low-intensity internal and external stimuli than individuals without ADHD. This interpretation is consistent with findings by Panagiotidi et al. (2020), who reported positive associations between sensory processing sensitivity and ADHD traits in a non-clinical student sample. Within the Research Domain Criteria (Insel et al., 2010) framework, these differences can be situated in the arousal domain, which conceptualizes arousal as a continuum of sensitivity to internal and external stimuli and highlights arousal modulation as a dynamic process linked to attentional allocation as well as motor, cognitive, and motivational activation (Feld & Feige, 2021). Because ADHD is frequently characterized by arousal dysregulation (Isaac et al., 2024), heightened orienting sensitivity may reflect two distinct mechanisms. First, it could represent a consequence of elevated baseline arousal, characterized by impaired top-down attenuation that results in increased sensitivity to irrelevant stimuli and disrupts task engagement. Alternatively, it may reflect a compensatory upregulation of sensory sensitivity, which serves to counteract hypoarousal by increasing engagement with internal or external stimuli, for instance through mind-wandering, spontaneous associative thinking, or scanning the environment for novel stimulation. Supporting this latter interpretation, Gomez et al. (2014) found that the orienting sensitivity subscale associative sensitivity, linked to spontaneous cognitive activity such as mind-wandering and internally driven thoughts, was associated with a general ADHD factor. Future research should examine orienting sensitivity-ADHD associations more closely to disentangle these mechanisms.

### Predictive Value of Temperament for ADHD Diagnosis

Regarding the prediction of an ADHD diagnosis (**H1.1** and **H1.2**), self-reported effortful control demonstrated a strong negative effect. A one-unit increase above the mean was associated with a 94% reduction in the odds of belonging to the ADHD group (see Table 4). This finding suggests that higher levels of self-reported effortful control are robustly linked to a substantially lower likelihood of an ADHD diagnosis. By contrast, a one-unit increase in negative affect was associated with a twofold increase in the odds of belonging to the ADHD group. Although this odds ratio may appear sizable, it is considered a small to moderate effect according to established benchmarks (Chen et al., 2010). The wide confidence interval (95% CI [1.05, 4.99]) further points to considerable estimation uncertainty, with the lower bound suggesting that the true effect may be minimal. Therefore, although elevated levels of self-reported negative affect are associated with adult ADHD, they are unlikely to be sufficient for predicting a diagnosis. The effect of surgency was not significant, which aligns with the absence of significant group differences observed for this temperament factor. When considered alongside research on ADHD in childhood and adolescence, our findings suggest that elevated reactive traits (surgency and negative affect), although considered central risk factors for the development of ADHD in childhood (Kerner auch Koerner et al., 2018; Martel et al., 2009), may be less relevant for the persistence of clinically significant ADHD symptoms into adulthood. In contrast, self-reported deficits in effortful control appear to be strongly associated with sustaining a clinical diagnosis of adult ADHD. However, this strong association does not imply that effortful control and ADHD should be equated. The substantially weaker association between both constructs in childhood (Kerner auch Koerner et al., 2018; Martel & Nigg, 2006), the unexplained variance remaining in adulthood (see results for binary logistic regression), and distinct clinical features of ADHD, such as impairments in motor function (Kaiser et al., 2015) and working memory (Alderson et al., 2013; Jones et al., 2016), highlight that ADHD is more than just an extreme expression of low effortful control (Nigg, 2006; Nigg et al., 2020; Rettew & McKee, 2005). Moreover, temperament and ADHD symptomatology are assessed through conceptually distinct instruments. The Adult Temperament Questionnaire captures dispositional self-regulatory tendencies, including voluntary control of attention and behavior in emotionally charged situations (Wiltink et al., 2006), distinguishing the effortful control factor from more general attentional and inhibitory impairments and daily problems, as measured by the Conners’ Adult ADHD Rating Scales (Christiansen et al., 2014). The substantial, yet incomplete, correlations between effortful control and ADHD symptoms suggest that affective self-regulatory traits contribute meaningfully to ADHD risk without fully defining the disorder. This aligns with dimensional models in developmental psychopathology, which conceptualize temperament as a vulnerability or protective factor interacting with other influences, rather than as a direct proxy for diagnostic outcomes (Nigg, 2006).

### Dual-Pathway Model of Temperament and ADHD in Adulthood

Neither the specific associations proclaimed by the dual-pathway model (Figure 1), nor the predictions of our compensatory model (Figure 2) were confirmed in our clinical adult sample. Contrary to expectations and to findings in younger populations (Kerner auch Koerner et al., 2018; Krieger et al., 2019; Martel et al., 2014), surgency did not contribute to the explanation of variance in self-reported hyperactive/impulsive symptoms. This likely reflects, at least in part, the previously mentioned factorial differences between the temperament construct in childhood/adolescence and adulthood (see discussion of missing group differences in surgency) and specifically, the absence of the activity level subscale from the surgency dimension in the Adult Temperament Questionnaire. While we could not find a clear rationale for this change in factor structure (Evans & Rothbart, 2007, 2009), one possible explanation is that motor activity becomes less relevant as a dispositional trait in adulthood, as it is increasingly shaped by contextual factors (e.g., occupation or lifestyle). In contrast, the structure of the Adult Temperament Questionnaire suggests a greater emphasis on affective and self-regulatory traits which may represent more stable and developmentally salient aspects of adult temperament (Evans & Rothbart, 2007). This change in factor structure would be consistent with typical findings showing that the general need for physical activity tends to decline with age, and that overt hyperactivity, commonly observed in childhood, often decreases during adolescence and adulthood, giving way to more internalized restlessness or subtler forms of arousal (Adler, 2004; Biederman et al., 2000). However, given the omission of motoric components, the surgency factor of the Adult Temperament Questionnaire may exhibit weaker associations with self-reported hyperactive/impulsive symptoms compared to measures used in children and adolescents. The negative yet non-significant association between surgency and self-reported inattentive symptoms in our data further suggests that the relationship between the adult surgency factor and ADHD warrants additional investigation.

The inclusion of effortful control strongly reduced the predictive value of negative affect for self-reported hyperactive/impulsive symptoms (CAARS scale DSM-HY/I *T*-scores; Table 5, Step 3). This indicates that both constructs share substantial variance, with effortful control emerging as the more salient predictor of adult ADHD symptomatology. This is in line with findings by Rabinovitz et al. (2016), who reported that the development of executive control, a construct associated with executive functions and effortful control and measured by a composite of different neuropsychological tests, partially explained the relationship between early anger/frustration and later ADHD symptom severity. Taken together with the strong correlation between negative affect and effortful control (see Table 2), our findings suggest that self-reported negative affect, as measured by the Adult Temperament Questionnaire, could partly reflect emotional dysregulation, which often accompanies adult ADHD, while other typical symptoms like externalized hyperactive behaviors tend to decline or may be suppressed in adulthood (Adler, 2004; Merkt et al., 2015; Retz et al., 2012).

The finding that age emerged as a positive predictor in the final model for hyperactive/impulsive symptoms can likely be attributed to a sampling effect, as the ADHD group was significantly older than the control group.

In line with our **H2.2**, self-reported effortful control significantly accounted for variance in both symptom domains of adult ADHD (see Tables 5 and 6), likely due to its integration of elements from both cold and hot executive functions (Kälin & Roebers, 2021; Tiego et al., 2020). While these findings differ from results reported in early childhood (Martel et al., 2009; Martel & Nigg, 2006), they are consistent with research in adolescence (Krieger et al., 2019; Wichstrøm et al., 2018) and adulthood (Gomez et al., 2014). This developmental shift may therefore reflect maturing processes of effortful control, which are assumed to parallel the structural and functional development of prefrontal brain regions throughout childhood into adolescence and young adulthood (Rothbart et al., 2006).

### Compensatory Model of ADHD and Temperament in Adulthood

Contrary to our expectations (**H3**), no moderating effect of effortful control on reactive traits was found (see Table 5, Step 5). This may partially be due to shared variance between negative affect and effortful control, to missing links between the adult surgency factor and self-reported hyperactive/impulsive symptoms and to insufficient statistical power. A post hoc power analysis indicated that the study was only powered to detect moderation effects with an *f^2^* of .06 or greater (1 − *β* = .80). Smaller effects may therefore have remained undetected. Previous studies have reported mixed results regarding the moderating role of effortful control (or related constructs) in different age groups (Kerner auch Koerner et al., 2018; Martel & Nigg, 2006; Rabinovitz et al., 2016). Such variability may possibly reflect age-related structural and functional brain maturation (Santens et al., 2020), or divergent emphases on specific components of reactive temperament. Furthermore, different methodological approaches could also explain inconsistent findings.

## Limitations

Several limitations should be considered, when interpreting the results of this study. First, the cross-sectional design limits causal inferences and may underestimate or bias moderation effects (Maxwell et al., 2011). Second, the clinical sample potentially consisted of “high functioning” adults with ADHD, who were diagnosed later in life and had high levels of education, which may affect generalizability of results. The control sample also mainly consisted of university students, which is why the observed group differences may reflect not only ADHD-specific effects, but also more general differences between clinical and non-clinical populations. Third, relying exclusively on self-report questionnaires may have introduced reporter bias, potentially compromising the validity of the data. Because self-reports capture subjective interpretations rather than objective indicators of temperament and ADHD symptomatology, they may produce systematic distortions and limit the generalizability of the findings. Moreover, reliance on a single informant may artificially inflate observed associations between variables due to shared method variance. Fourth, while group assignments (relevant to **H1.1**/**H1.2**) were primarily based on a multimodal diagnostic process, including clinical interviews, self-/informant reports, and behavioral observations, the Conners’ Adult ADHD Rating Scales were also incorporated into this process. Using them as both a diagnostic component and an outcome measure carries a risk of circularity, which may inflate observed group differences, despite partial mitigation through the use of independent diagnostic sources. Lastly, item overlap between effortful control and ADHD scales may account for some of the large effect sizes observed in this study. We have attempted to address this issue by removing items with literal overlap with the DSM-5 criteria from the Adult Temperament Questionnaire. However, this method leaves open the question of what degree of semantic overlap is acceptable (Sanson et al., 1990; Wichstrøm et al., 2018). Although Nigg (2006) points out that effect sizes remained comparable before and after the removal of overlapping items in previous studies, the degree of methodological conservatism in handling item overlap can still influence results. Therefore, the effect sizes related to the effortful control factor should be interpreted with caution.

## Implications

The limitations discussed above suggest several implications for future study designs. First, a longitudinal approach across various developmental stages is needed to examine temperamental interaction effects over time (Maxwell et al., 2011). Second, future research should employ more representative sampling of adults with ADHD by including both late-onset and childhood-onset cases. Including psychiatric control groups or applying matched sampling procedures could further help to isolate temperament-ADHD associations. Third, adopting multi-method approaches that combine multi-informant assessments with performance-based measures would help to distinguish true trait covariance from shared method effects (Nigg, 2006). This approach would simultaneously address the methodological limitations of mono-informant method, reporter-based bias, and item overlap. However, performance-based assessments should complement self-reports rather than replace them, as both capture different levels of analysis and underlying processes (Soto et al., 2020; Toplak et al., 2013). Given that effortful control includes motivational and emotional aspects of self-regulation, developing ecologically valid performance-based assessments for adults remains a key challenge. Additionally, while there are well-validated informant-report measures for children and adolescents (Ellis & Rothbart, 2001; Putnam & Rothbart, 2006), to our knowledge, no comparable measure has yet been established for adults. The development and validation of such an instrument is therefore a central objective for future research. Fourth, to rule out the potential risk of circularity, future studies should use different ADHD self-report measures for diagnosis and outcome measurement.

Beyond methodological refinements, the present findings raise broader theoretical questions about the developmental continuity and structural coherence of temperament traits into adulthood. Rothbart and colleagues propose that temperament, while evolving through maturation and environmental influences, retains relative stability as a biological foundation of personality (Evans & Rothbart, 2007; Rothbart & Bates, 2006). Within this framework, temperament is best understood as exhibiting heterotypic stability, whereby core dispositions remain stable, but their behavioral expressions shift across developmental stages. Accordingly, the number and type of traits assessed in questionnaires may need to be adapted across the lifespan to ensure age-appropriate measurement (Dyson et al., 2015). Empirical findings support this perspective: Putnam et al. (2001) demonstrated that temperament-related behaviors undergo substantial reorganization from early childhood to adolescence, and differences between the Adult Temperament Questionnaire and the Early Adolescent Temperament Questionnaire suggest that such shifts, particularly in surgency, extend into adulthood. At the same time, structural changes may partly reflect methodological artifacts, such as sampling variation or analytic decisions. Longitudinal multi-method, multi-informant studies spanning childhood to adulthood are therefore needed to determine whether observed structural differences represent true developmental change or methodological variability. Finally, the strong correlation between negative affect and effortful control indicates substantial overlap at the observed-score level. This overlap highlights the need for future studies to examine more closely whether negative affect and effortful control represent separable dimensions at the latent level, for example through factor-analytic approaches and tests of measurement invariance across clinical and non-clinical groups.

With regard to clinical application, temperament traits, as in childhood, remain clinically informative in adulthood and may help delineate subgroups within adult ADHD populations. Specifically, temperament-based assessments may support the development of more personalized diagnostic and treatment approaches. In this context, studies by Karalunas et al. (2019) and Goh et al. (2020) have shown that children with high irritability and poor cognitive functioning tend to follow more severe and persistent ADHD trajectories. These findings raise the question of whether such temperament-based subgroupings could also prove useful in adulthood. Our study provides a first step in addressing this gap by demonstrating that similar temperamental characteristics are meaningfully associated with adult ADHD symptoms. Our findings highlight the reciprocal relationship between self-reported effortful control and negative affect, suggesting that deficits in self-regulation may amplify negative affective states, which in turn may impair access to effective regulation strategies. This dynamic supports the view that emotional dysregulation is a core component of ADHD rather than a mere comorbidity (Nigg, 2022; Retz et al., 2012). Integrating temperament assessments into diagnostic and therapeutic frameworks could thus enhance clinicians’ ability to identify emotional and regulatory vulnerabilities in adults with ADHD. Moreover, the finding suggests a potential feedback loop that could be leveraged through targeted interventions. Strengthening effortful control skills may not only improve attentional and behavioral functioning, but also reduce psychological distress by addressing underlying temperamental mechanisms linked to negative affectivity, thereby facilitating better access to self-regulatory abilities.

## Conclusion

Taken together, our findings contribute to a cross-age understanding of temperament-ADHD associations by providing novel data on adults with a clinical ADHD diagnosis. While childhood studies have consistently identified reactive traits such as surgency and negative affect as key vulnerability factors for the development of ADHD (e.g., Kerner auch Koerner et al., 2018; Martel et al., 2009), our results highlight self-perceived deficits in effortful control as the primary temperamental predictor of ADHD persistence into adulthood. This pattern questions assumptions from dual-pathway models that propose largely independent roles for reactive traits and self-regulatory deficits in ADHD symptomatology. Assessing temperament across the lifespan may thus help clarify early risk profiles and inform our understanding of phenotypic heterogeneity and maintaining mechanisms in adulthood (Nigg et al., 2020). Importantly, the association between the adult surgency factor and adult ADHD appears to diverge from patterns typically observed in children. This highlights the need for further longitudinal and multi-method research to examine how temperament traits evolve and interact with psychopathology across developmental stages.

Overall, our findings suggest that temperament measures, particularly those assessing regulatory capacity, may hold potential for enhancing clinical understanding of adult ADHD and could help guide more individualized diagnostic and therapeutic approaches.

## Appendix

**Table A1.** ATQ Items Removed From Analyses Due to Direct Overlap in Content With CAARS Items and DSM-5 Criteria.

ATQ-No.	ATQ-Item	ATQ-Scale	CAARS-No., Scale	DSM-5 Criteria
11	Can sit still, even when energized.	EC (IC)	21, DSM-HY/I	*Trouble staying still:* Often leaves seat in situations when remaining seated is expected (e.g., in the office, or during movies) *Restlessness*: Desire to move about when it’s inappropriate or consistent feelings of restlessness that are difficult to control
29R	Easily distracted when trying to focus.	EC (AtC)	24, DSM-IA; 64, DSM-IA	*Diminished attention span:* Often has difficulty sustaining attention in tasks or play activities (e.g., has difficulty remaining focused during lectures, conversations, or lengthy reading) *Easily distracted*: Is often easily distracted by things in the environment or unrelated thoughts.
35	Can easily shift attention back after interruptions.	EC (AtC)	24, DSM-IA	*Easily distracted:* Is often easily distracted by things in the environment or unrelated thoughts.
43	Can easily resist talking out of turn, even when excited.	EC (IC)	58, DSM-HY/I	*Impulsively blurting things out:* Often blurts out an answer before a question has been completed, completes people’s sentences, or cannot wait to speak in a conversation.
55	Finish things before they are due.	EC (AcC)	60, DSM-IA; 65, DSM-IA	*Failing to complete tasks:* Often does not follow through on instructions and fails to finish schoolwork, chores, or duties in the workplace (e.g., starts tasks but quickly loses focus and is easily sidetracked). *Poor organizational skills:* Often has difficulty organizing tasks and activities (e.g., difficulty managing sequential tasks; difficulty keeping materials and belongings in order; messy, disorganized work; has poor time management; fails to meet deadlines).
31	Become agitated when having to sit and wait.	NA (Fr)	22, DSM-HY/I	*Trouble waiting your turn:* Often has difficulty waiting your turn, such as waiting in line.
6R	Rarely annoyed in slow-moving lines.	NA (Fr)	22, DSM-HY/I	*Trouble waiting your turn:* Often has difficulty waiting your turn, such as waiting in line.
19	Like talking a lot.	SUR (Soc)	9, DSM-HY/I	*Often talks excessively:* Excessively chatty, sometimes unaware of the effect it has on others.

*Note.* The full display of ATQ and CAARS items is not possible due to copyright restrictions. No. = item number; ATQ = Adult Temperament Questionnaire; CAARS = Conners’ Adult ADHD Rating Scales; DSM-IA = DSM inattention symptoms; DSM-HY/I = DSM hyperactivity/impulsivity symptoms; EC (AtC) = Effortful Control (attentional control); EC (IC) = Effortful Control (inhibitory control); NA (FR) = Negative Affect (frustration); SUR (Soc) = Surgency (sociability).

**Table A2.** ATQ Items With Potential Overlap in Content With CAARS Items and DSM-5 Criteria and Reasons for Non-Removal From Analyses.

ATQ-no.	ATQ-item	ATQ-Scale	CAARS-No., Scale	DSM-5 Criteria	Reason for non-removal
47	Get to work immediately, if thinking of something to do.	EC (AcC)	—	—	No direct overlap with DSM-5 criterion or CAARS-DSM scale
40R	Hard to focus when distressed.	EC (AtC)	24, DSM-IA; 64, DSM-IA	*Diminished attention span:* Often has difficulty sustaining attention in tasks or play activities (e.g., has difficulty remaining focused during lectures, conversations, or lengthy reading) *Easily distracted*: Is often easily distracted by things in the environment or unrelated thoughts.	ATQ item incorporates emotional aspect (distress)
50R	Hard to focus when excited.	EC (AtC)	24, DSM-IA; 64, DSM-IA	*Diminished attention span:* Often has difficulty sustaining attention in tasks or play activities (e.g., has difficulty remaining focused during lectures, conversations, or lengthy reading) *Easily distracted*: Is often easily distracted by things in the environment or unrelated thoughts.	ATQ item incorporates emotional aspect (excitement)
5R	Hard to alternate between two different tasks.	EC (AtC)	—	—	No direct overlap with DSM-5 criterion or CAARS-DSM scale: measurement of task switching (ATQ) vs. doing multiple things at once (CAARS)
8R	Do not follow through with own plans.	EC (AcC)	—	*Lacks follow-through:* Often does not follow through on instructions and fails to finish schoolwork, chores, or duties in the workplace, quickly losing focus and getting sidetracked.	No direct overlap with CAARS-DSM scale and measurement of different aspects: Failure to execute one’s own existing plans (ATQ) vs. general problems completing a task even when others have planned it for you (DSM-5)
27	Able to work on tasks, even without motivation.	EC (AcC)	48, DSM-IA	*Avoid tasks requiring concentration:* Often avoids, dislikes, or is reluctant to engage in tasks that require sustained mental effort (e.g., preparing reports, completing forms, reviewing lengthy papers)	Measurement of different aspects: EC item incorporates motivational aspect (ATQ) vs. general aversion to cognitive effort (CAARS/DSM-5)
2R	Often late for appointments.	EC (AcC)	65, DSM-IA	*Disorganized:* Often has difficulty organizing tasks and activities, including managing sequential tasks, keeping belongings in order, and having poor time management and meeting deadlines.	No direct overlap with DSM-5 criterion or CAARS-DSM scale: ATQ item measures certain aspect of poor organizational skills
72R	Avoid situations if afraid of outcome.	EC (AcC)	—	—	ATQ item incorporates emotional aspect (fear)
15	Can continue tasks, even when unwilling.	EC (AcC)	60, DSM-IA	*Lacks follow-through:* Often does not follow through on instructions and fails to finish schoolwork, chores, or duties in the workplace (e.g., starts tasks but quickly loses focus and is easily sidetracked)	Measurement of different aspects: EC item incorporates motivational aspect (ATQ) vs. general distractibility (CAARS/DSM-5)
26	Can hold back laughter if not appropriate.	EC (IC)	—	—	ATQ item describes impulsivity in an emotional/motivational context
53R	Hard to resist cravings for food or drinks.	EC (IC)	—	—	ATQ item describes impulsivity in an emotional/motivational context
60R	Hard to resist jumping into exciting things.	EC (IC)	—	—	ATQ item describes impulsivity in an emotional/motivational context
76	Can inhibit inappropriate fun behavior.	EC (IC)	—	—	ATQ item describes impulsivity in an emotional/motivational context
63R	Hard to resist buying attractive items.	EC (IC)	—	—	ATQ item describes impulsivity in an emotional/motivational context
38R	Usually a patient person.	NA (Fr)	22, DSM-HY/I	*Trouble waiting your turn:* Often has difficulty waiting your turn, such as waiting in line.	No direct overlap with DSM-5 criterion or CAARS-DSM scale: difference between waiting your term/patients in general
58R	Remain calm when things don’t go smoothly.	NA (Fr)	—	—	No direct overlap with DSM-5 criterion or CAARS-DSM scale
48	Easily frustrated or irritated.	NA (Fr)	—	—	No direct overlap with DSM-5 criterion or CAARS-DSM scale
73	Enjoy not having to think in conversations.	SUR (HiP)	—	*Often talks excessively:* Excessively chatty, sometimes unaware of the effect it has on others.	ATQ item incorporates emotional aspect (enjoyment)

*Note.* The full display of ATQ and CAARS items is not possible due to copyright restrictions. No. = item number; ATQ = Adult Temperament Questionnaire; CAARS = Conners’ Adult ADHD Rating Scales; DSM-IA = DSM inattention symptoms; DSM-HY/I = DSM hyperactivity/impulsivity symptoms; EC (AtC) = Effortful Control (attentional control ); EC (AcC) = Effortful Control (activation control); EC (IC) = Effortful Control (inhibitory control); NA (FR) = Negative Affect (frustration); SUR (Soc) = Surgency (sociability); SUR (HiP) = Surgency (high-intensity pleasure).

**Table A3.** Adjusted and Unadjusted Means (and Effect Sizes) for Temperament Factors by Group Status.

	*n*	Unadjusted means		Adjusted means		Partial *η*^2^
Variable		*M*	*SD*	*M*	*SE*	
Surgency						
ADHD group	75	4.48	.88	4.51	.10	.001
Control group	78	4.56	.81	4.53	.10	
Negative affect						
ADHD group	75	4.39	.66	4.41	.09	.327**
Control group	78	3.39	.81	3.36	.09	
Effortful control						
ADHD group	75	3.09	.70	3.11	.09	.522**
Control group	78	4.77	.82	4.75	.09	
Orienting sensitivity						
ADHD group	75	4.76	.97	4.80	.10	.051*
Control group	78	4.44	.79	4.39	.10	

*Note*. Adjusted means are controlled for age and educational level. Educational level was dichotomized (0 = intermediate secondary school certificate or lower; 1 = university entrance degree). Effect sizes from the ANCOVA did not differ significantly from those obtained via independent-samples *t*-tests. *M* = mean; *SD* = standard deviation; *SE* = standard error; partial *η^2^* = partial eta-squared for group difference; **p* < .05; ***p* < .001.
